# Pleiotropic effect of sodium-glucose cotransporter 2 inhibitors on blood pressure

**DOI:** 10.3389/fcvm.2022.1086672

**Published:** 2022-12-20

**Authors:** Ting-Wei Kao, Chin-Chou Huang

**Affiliations:** ^1^Department of Internal Medicine, National Taiwan University Hospital, Taipei, Taiwan; ^2^Department of Medical Education, Taipei Veterans General Hospital, Taipei, Taiwan; ^3^Division of Cardiology, Department of Medicine, Taipei Veterans General Hospital, Taipei, Taiwan; ^4^School of Medicine, National Yang Ming Chiao Tung University, Taipei, Taiwan; ^5^Institute of Pharmacology, National Yang Ming Chiao Tung University, Taipei, Taiwan; ^6^Cardiovascular Research Center, National Yang Ming Chiao Tung University, Taipei, Taiwan

**Keywords:** SGLT2 inhibitor, blood pressure, pleiotropy, diabetes, heart failure, hypertension

## Abstract

Sodium-glucose cotransporter 2 (SGLT2) inhibitors have been incorporated as guideline-directed medical therapy for heart failure with reduced ejection fraction. Recent trials clearly established the efficacy of SGLT2 inhibitors on cardiac remodeling while preventing renal function decline in patients with or without diabetes mellitus. Blood pressure reduction during SGLT2 inhibitors use has been proposed through pleiotropic pathways and as a potential contributor that translates to cardiovascular benefits. The mechanisms underlying this decrease in blood pressure are not simply glycemic control. Orchestrating fluid status, modulation of sodium content and renin-angiotensin-activation system, anti-fibrosis and anti-inflammatory effect, ameliorating the characteristics of metabolic syndrome, as well as restoration of circadian rhythm all contributed to the BP lowering effect by SGLT2 inhibitors. Although SGLT2 inhibitors has not been demonstrated as anti-hypertensive agents thus far, their effects on BP alteration are clinically significant. In this review, we revisited the evidence correlating SGLT2 inhibitor use with blood pressure level. Future research directions will focus on the signaling pathway of SGLT2 inhibitors for fluid removal, atherosclerosis, vasoconstriction, and eventually hypertension.

## 1 Background

Over the past decade, sodium-glucose cotransporter 2 (SGLT2) inhibitors have been indicated for the management of diabetes as well as treating heart failure with reduced ejection fraction. An accumulating body of evidence has also suggested that the administration of SGLT2 inhibitors was associated with better overall survival as well as cardiovascular and renal prognosis (1). The benefit of cardiovascular outcomes with SGLT2 inhibitors was mainly driven by a reduction in hospitalization for heart failure and cardiovascular death. However, the mechanism of action and hemodynamic impact of such medications have remained largely elusive. Preliminary concepts regarding SGLT2 inhibitors suggest it is a diuretic with stronger potency. It also has natriuretic effects. The efficacy was shown to be consistent in the presence or absence of diabetes. These investigations into the pleiotropic effects of SGLT2 inhibitors for improved cardiovascular outcomes show a surprising benefit. Blood pressure (BP) is an indicator of hemodynamic effects and was among the endpoints remarkably altered by introducing SGLT2 inhibitors as demonstrated in observational studies. Better delineation on BP impact of SGLT2 inhibitor will facilitate its use in patients with difference baseline BP level.

The correlation between SGLT2 attenuation and the extent of BP alteration is a knowledge gap. Understanding how the presence of comorbidities affects the hemodynamic response requires more than observational studies. The aim of this review is therefore to revisit the clinical performance of SGLT2 inhibitors in patients with various demographic backgrounds and then interrogate the underlying spectrum of mechanisms impacting BP by SGLT2 inhibitors.

## 2 BP reductions in the literature

### 2.1 Patients with diabetes

The extent of BP reduction varies as a function of patient characteristics and type of SGLT2 inhibitor administered (Table 1). Initial studies focused on the effects in patients with diabetes including pooling analysis of four phase 3 studies of SGLT2 inhibitors; the inclusion criteria did not mandate the presence of other comorbidities. The results showed that 100 and 300 mg canagliflozin daily was associated with 4.3 and 5.0 mmHg reduction in systolic BP (2).

**TABLE 1:** Sensitivity analyses of the associations between genetically predicted iron status and mental disorders.

References	Study type	SGLT2 inhibitor	Patient group	BP measurement	BP reduction (mmHg)
**Patients with DM**					
Weir et al. (2)	MA	Canagliflozin 100 mg/d, 300 mg/d	DM	Office BP	100 mg/d: SBP 4.3, DBP 2.5 300 mg/d: SBP 5.0, DBP 2.4
Zinman et al. (3)	RCT	Empagliflozin 10 mg/d, 25 mg/d	DM and established cardiovascular disease	Office BP	SBP 5, DBP 3
Neal et al. (4)	RCT	Canagliflozin 100 mg/d, optional increase to 300 mg/d	DM and high cardiovascular risk	Office BP	SBP 3.93, DBP 1.39
Wiviott et al. (5)	RCT	Dapagliflozin 10 mg/d	DM and at risk for atherosclerotic cardiovascular disease	Office BP	SBP 2.7, DBP 0.7
Tsapas et al. (6)	MA	Empagliflozin, canagliflozin, dapagliflozin, ertugliflozin	DM	Office BP	SBP 2.89, DBP 1.44
**Patients with DM and specified renal function**					
Tikkanen et al. (8)	RCT	Empagliflozin 10 mg/d or 25 mg/d	DM, intact renal function	24-h ambulatory BP	10 mg/d: ASBP 3.44, ADBP 1.36 25 mg/d: ASBP 4.16, ADBP 1.72
Perkovic et al. (7)	RCT	Canagliflozin 100 mg/d	DM and CKD	Office BP	SBP 3.3, DBP 0.95
**Patients with DM and hypertension**					
Weber et al. (12)	Double-blind, placebo-controlled	Dapagliflozin 10 mg/d	DM and hypertension	Office BP	SBP 11.9
Kario et al. (11)	Double-blind, parallel	Empagliflozin 10 mg/d	DM and uncontrolled nocturnal hypertension	Office BP, home BP, 24-h ambulatory BP	SBP daytime 9.5, nighttime 6.3, 24 ASBP 7.7, morning home 7.5, office 8.6
Ferdinand et al. (13)	Randomized open-label trial	Empagliflozin 10 mg/d, then titrated to 25 mg/d	DM and hypertension (African American)	Office BP, ambulatory BP	Office SBP: 10.3, ASBP 10.3, nighttime 6.7
**Patients with heart failure**					
McMurray et al. (15)	RCT	Dapagliflozin 10 mg/d	HF (LVEF < 40%)	Office BP	SBP 1.27
Packer et al. (16)	RCT	Empagliflozin 10 mg/d	HF (LVEF < 40%)	Office BP	SBP 1–2
Anker et al. (17)	RCT	Empagliflozin 10 mg/d	HF (LVEF > 40%)	Office BP	SBP 1.8
Nassif et al. (18)	RCT	Dapagliflozin 10 mg/d	HF (LVEF ≥ 45%)	Office BP	SBP 1
Solomon et al. (19)	RCT	Dapagliflozin 10 mg/d	HF (LVEF > 40%)	Not reported	
Li et al. (20)	MA	Not specified	HF	Office BP	SBP: 1.68, DBP: 1.06

ADBP, ambulatory diastolic blood pressure; ASBP, ambulatory systolic blood pressure; BP, blood pressure; CKD, chronic kidney disease; DBP, diastolic blood pressure; DM, diabetes mellitus; HF, heart failure; LVEF, left ventricular ejection fraction; MA, meta-analysis; RCT, randomized control trial; SBP, systolic blood pressure; SGLT2, sodium-glucose cotransporter 2.

The enrolled subjects of initial landmark trials regarding SGLT2 inhibitors were those with diabetes and high cardiovascular risk. In the EMPA-REG OUTCOME (Empagliflozin Cardiovascular Outcome Event Trial in Type 2 Diabetes Mellitus Patients) trial, patients with diabetes and cardiovascular disease risk factors were enrolled to receive either empagliflozin or placebo. The reductions in systolic and diastolic BP were 5 and 3 mmHg, respectively, after 16 weeks after administration (3). The CANVAS (Canagliflozin Cardiovascular Assessment Study) trial gave canagliflozin to patients with similar clinical backgrounds: The systolic and diastolic BP reductions were 3.93 and 1.39 mmHg, respectively (4). Dapagliflozin was given in the same clinical setting as the DECLARE-TIMI 58 (Dapagliflozin Effect on CardiovasculAR Events) trial. The results showed changes in systolic and diastolic BP of 2.7 and 0.7 mmHg, respectively (5). A recent meta-analysis of 64 studies showed a systolic and diastolic BP reduction of 2.89 and 1.44 mmHg, respectively, in a diabetic population (6).

Subsequent studies have focused on the renal function of patients with type 2 diabetes. The CREDENCE (Canagliflozin and Renal Events in Diabetes with Established Nephropathy Clinical Evaluation) trial introduced canagliflozin in subjects with diabetes and albuminuria chronic kidney disease. Treatment was associated with 3.3 and 0.95 mmHg BP reductions (7). The EMPA-REG BP (Empagliflozin Reduces Blood Pressure in Patients with Type 2 Diabetes and Hypertension) trial studied patients with diabetes, intact renal function at baseline, and initially normotensive or stage I hypertension. The results suggested that daily 10 mg and 25 mg empagliflozin was associated with 3.44/4.16 mmHg reductions in ambulatory systolic BP and 1.36/1.72 mmHg reductions in ambulatory diastolic BP at week 12. The effect was preserved even in patients who underwent other antihypertensive medications at baseline. More individuals in the SGLT2 inhibitor arm reached systolic BP target at <130 mmHg vs. those under placebo (8).

Early literature has recognized hypertension both as a cause and as a consequence of kidney dysfunction (9), and thus concomitant use of other medications that might alter kidney function is important for the performance of SGLT2 attenuation. No direct evidence is thus far available to interrogate the interplay, whereas the abrogation of changes in estimated glomerular filtration rate might provide important clues. Kitumura et al. retrospectively suggested that background uses of metformin attenuated the decline of estimated glomerular filtration rate after SGLT2 inhibitor use, whereas concomitant administration of renin-angiotensin activation system (RAAS) inhibitors diminished such effect. Baseline administration of insulin, dipeptidyl peptidase 4 inhibitors, β blockers, and calcium channel blocker had no effect (10).

Patients with diabetes and hypertension at baseline have a greater BP reduction by SGLT2 inhibitors. The SACRA (SGLT-2i and ARB Combination Therapy in Patients with T2DM and Nocturnal Hypertension) study attempted to investigate the role of SGLT2 inhibitors in diabetes accompanying refractory nocturnal hypertension. The results showed a 7.7/2.9 mmHg reduction in systolic and diastolic ambulatory BP. Nevertheless, the average decrease in nocturnal BP was less obvious at 4.3/1.6 mmHg (11). Weber et al. investigated 449 individuals with diabetes and hypertension and proposed a SGLT2 attenuation that decreased systolic BP with an average of 4.28 mmHg. Interestingly, patients on β blockers or calcium channel blockers benefited from more pronounced BP reduction (12). From the perspective of race, Ferdinand et al. enrolled 154 African American patients with diabetes and hypertension and showed more pronounced systolic and diastolic BP reductions at 7.4 and 4.3 mmHg, respectively (13).

### 2.2 Patients with heart failure

Other concomitant studies focused on populations with heart failure to identify the BP effect of SGLT2 inhibitors (Table 1). Although promising in terms of improving cardiovascular and renal prognosis irrespective of diabetic status in such population (14), BP alterations via SGLT2 inhibitors were less pronounced. The DAPA-HF (The Study to Evaluate the Effect of Dapagliflozin on the Incidence of Worsening Heart Failure or Cardiovascular Death in Patients with Chronic Heart Failure) trial specifically enrolled subjects with left ventricular ejection fraction values less than 40%. The results demonstrated a 1.27 mmHg reduction of systolic BP by 10 mg daily dapagliflozin (15). Similarly, the EMPA-Reduced (The Empagliflozin Outcome Trial in Patients with Chronic Heart Failure With Reduced Ejection Fraction) trial targeted the same patient group and proposed only a 1–2 mmHg decrease of systolic BP. The BP lowering effect was further diminished during SGLT2 treatment after 4 weeks (16).

The effect was comparative under the setting of heart failure with preserved ejection fraction. The EMPEROR-Preserved (EMPagliflozin outcomE tRial in Patients With chrOnic heaRt Failure with Preserved Ejection Fraction) trial excluded individuals with systolic hypertension greater than 180 mmHg and hypotension less than 100 mmHg at randomization. The results suggested that empagliflozin was associated with only a 1.8 mmHg decrease in systolic BP (17). In the PRESERVED-HF (Dapagliflozin in PRESERVED Ejection Fraction Heart Failure) trial, however, there was no significant change in BP by dapagliflozin and placebo (18). As for DELIVER (Dapagliflozin Evaluation to Improve the Lives of Patients With Preserved Ejection Fraction Heart Failure) trial in which the cardioprotective effect by dapagliflozin was assessed in patients with an ejection fraction greater than 40%, individuals with a systolic BP less than 95 mmHg were excluded. No data of BP alteration during the follow-up period was available (19). Recent meta-analysis pooling of 16 randomized control trials regarding patients with heart failure in general indicated that SGLT2 inhibition was associated with modest but statistically significant reductions in systolic BP (1.68 mmHg) but not diastolic BP (1.06 mmHg) (20). Unlike diuretics that resulted in immediate and sustained BP reduction, the BP-lowing effect of SGLT2 inhibition in individuals with heart failure was minimal and transient, thus implying that the clinical benefit might stem from the anatomical and functional remodeling of the cardiovascular system secondary to other pleiotropic effects. The following section revisits the pharmaceutical effects of SGLT2 inhibitors.

## 3 Pleiotropic mechanisms

### 3.1 Orchestrate fluid status

Several mechanisms have been proposed to orchestrate BP under SGLT2 inhibitor use. First, pronounced alterations in fluid status are the most prominent pleiotropic effect. Reduction of intravascular fluid by SGTL2 inhibitors is a straightforward mechanism to control BP. Beyond diuretic effect, SGLT2 inhibitors antagonize fluid retention not only with a stronger potency but also conjunctionally exert a natriuretic effect. A pharmacodynamic study showed that canagliflozin led to higher urine sodium levels at earlier phases of treatment (21). This resulted from the attenuated reabsorption of sodium over proximal renal tubules and facilitated sodium delivery to macula densa. A randomized controlled trial suggested that administration of dapagliflozin was associated with a 7% decrease in plasma volume in the presence of diabetes—this directly contributed to a 3.3 mmHg systolic BP reduction (22). The EMPAG-HF trial demonstrated that the introduction of empagliflozin in addition to standard loop diuretics significantly increased renal output without compromising renal function in the management of acute decompensatory heart failure (23). Another study proposed treating patients sustaining heart failure with reduced ejection fraction with SGLT2 inhibitors was associated with reduced dose of loop diuretics to orchestrate fluid status (24). There was nevertheless no significant difference in the dose of loop diuretics between groups in DAPA-HF trial. Besides, SGLT2 inhibitors were associated with reduction in cardiovascular mortality and hospitalization for heart failure, which have not been demonstrated by loop diuretics. Further pleiotropic effects warranted investigations.

### 3.2 Sodium effect

Sodium-glucose cotransporter 2 inhibitors have been established to decrease circulating sodium concentrations through various effects on renal physiology. Both osmotic diuresis and natriuresis have contributed to renal excretion of sodium and therefore can ameliorate salt-sensitive hypertension (25). In a large meta-analysis of 31,949 subjects from 21 trials, subjects who consumed salt substitutes were correlated with a greater BP reduction (26). However, the BP-lowering effect was argued to be secondary not only to reduced sodium levels but also to hyperkalemia as is common in the pooled analysis.

Sodium-glucose cotransporter 2 inhibitors have been postulated to increase serum potassium levels in patients with diabetes and intact renal function. In a rodent model, the increased expression of intrarenal angiotensinogen was circumvented by the introduction of canagliflozin (27). However, a *post hoc* analysis in the CREDENCE trial demonstrated that administering SGLT2 inhibitors on top of RAAS inhibition intriguingly reduced the risk of hyperkalemia in individuals with diabetes and chronic kidney disease (28). A meta-analysis involving 49,875 individuals confirmed this finding (29). The recent landmark SsaSS (Salt Substitute and Stroke Study) trial further extended the benefit of lower sodium levels to better neurological outcomes and all-cause mortality (30). Future studies are needed to correlate the effect of SGLT2 inhibitors on electrolytes with cardiovascular outcomes.

### 3.3 RAAS modulation

Renin-angiotensin activation system plays another central role in the regulation of BP. Since thus far there is a paucity of direct measurement regarding intrarenal angiotensin II level, the effects of SGLT2 inhibitors on RAAS remained undetermined (31). Differences in the class effect might result from the heterogeneous study design and interactions with other pleiotropic mechanisms (32). Administering SGLT2 inhibitors on rats with diabetes was demonstrated to remarkably decrease urinary excretion of angiotensinogen and reflects intrarenal RAAS activity (33). Both in animal and human studies, the long-term treatment of dapagliflozin in OLETF (Otsuka Long Evans Tokushima fatty) rats and individuals with diabetes (33) resulted in greater plasma renin activity but intact serum aldosterone concentration. However, the alteration of plasma renin activity was only observed in the acute phase after SGLT2 inhibition (34). Evidence also exited suggesting SGLT2 inhibitors use was not associated with the activation of RAAS as loop diuretics (35). Together, diuresis by greater glucose concentration in tubule and mild natriuresis contributed to negative water and sodium balance, mildly depleted intravascular volume, and ultimately the BP lowering effect.

### 3.4 Anti-fibrosis and anti-inflammation

Anti-inflammatory effects are a hallmark pleiotropic effect of SGLT2 inhibitors. Mechanistically, the nitric oxide-cyclic guanosine monophosphate-protein kinase G pathway is known to produce vasodilation and thus BP reduction. As demonstrated in a porcine model, SGLT2 inhibition led to the activation of such signaling and promoted the phosphorylation of endothelial nitric oxide synthase (36). Second, inflammation and the activation of RAAS are closely intertwined. The anti-inflammatory effect attenuated the activity of RAAS through down-regulation of vascular endothelial growth factors and prostaglandin (37). In a rodent model, introducing empagliflozin remarkably antagonized the activation of nuclear factor-κB and mitogen-activated protein kinase. This effect blockaded both the local infiltration of macrophages and angiogenesis (38). The effect was also reflected by the viscoelasticity of the vessel wall. Applying pulse wave velocity to exemplify vasculature rigidity, Cherney et al. illustrated that administering empagliflozin was associated with reduced arterial stiffness regardless of glycemic status in young patients with type 1 diabetes (39). Besides, accumulating hypothesis-generating studies recognized the role of fat cells in inflammation. The epicardial adipocytes regulate the paracrine to modulate inflammatory response, which is promoted by leptin and ameliorated by adiponectin. Obesity, insulin resistance, and diabetes were proposed to orchestrate the adipokine level and alter inflammation. Anti-inflammatory effects by the administration of SGLT2 inhibitors were proposed through decreased levels of leptin, interleukin-6, plasminogen activator inhibitor-1, as well as ectopic epicardial fat (40).

### 3.5 Ameliorating metabolic syndrome

Metabolic syndrome is characterized by the presence of central obesity, elevated BP, high serum glucose level, hypertriglyceridemia, and low high-density lipoprotein concentration (41). Administration of SGLT2 inhibitors was demonstrated to alter these clinical indexes of metabolic syndrome and collectively led to the orchestration of BP level. Prior work showed that SGLT2 inhibitors led to body weight reductions in patients with diabetes (42). The main mechanism was considered to be the loss of visceral and subcutaneous adipose tissue. A recent meta-analysis pooling of 116 randomized trials further suggested significant body weight reductions at 1.79 kg. This weight loss was associated with SGLT2 inhibitors particularly in obese subjects regardless of diabetic status (43). After an extensive follow-up period, the legacy effect of body weight reductions was considered durable. Relevant mechanisms included better glycemic control and insulin sensitivity. Therefore, the interpretations should be prudently considered as one of the pleiotropic effects acting synergistically with other antihypertensive effects of SGLT2 inhibition.

The metabolism of circulating lipoprotein is pivotal in the pathogenesis of atherosclerosis and hypertension. A detailed summary of how SGLT2 inhibitors reduce triglycerides has been reviewed elsewhere (44). Briefly, SGLT2 inhibitors were associated with normalized diacylglycerol O-acyltransferase 2 mRNA, increased peroxisome proliferator-activated receptor-α but reduced receptor-γ, elevated fibroblast growth factor 21, and upregulated cluster of differentiation 36. Mechanistically, this reduction in triglyceride is nevertheless associated with an elevation of serum low-density lipoprotein (LDL) levels as well potentially through antagonized cholesteryl ester transfer protein activity as well as accelerated very-low-density lipoprotein-to-LDL conversion. A rodent model recapitulating diabetes concurred that SGLT2 inhibitors attenuate LDL clearance and promote serum accumulation (45). Recent meta-analysis pooling of 48 randomized controlled trials illustrated that SGLT2 inhibitors reduced triglyceride levels but increased LDL, high-density lipoprotein, and total cholesterol (46). The impacts on the lipid metabolism of SGLT2 inhibitors is via altered remodeling of arterial vasculature BP. Future studies are warranted to further clarify the hemodynamic consequences. Individuals with metabolic syndrome are frequently complicated with hyperuricemia. Reductions in serum uric acid were proposed to also be relevant to hemodynamic effects. The literature suggests that the accumulation of uric acid potentiated the risk of cardiorenal complications. Zhao et al. pooled 34,941 individuals with diabetes and suggested that the administration of SGLT2 inhibitors was associated with rapid-onset reductions in uric acid (47). By retrospectively analyzing two randomized trials (Renoprotective Effects of Dapagliflozin in Type 2 Diabetes study, Uric Acid Excretion study), the effect of uric acid excretion under dapagliflozin and empagliflozin was proposed to be associated with urinary glucose excretion (48). However, it remains unclear whether reduced uric levels translate to cardiovascular benefits. Based on the outcomes of introducing xanthine oxidase inhibitors, managing hyperuricemia can be effective at abating hypertension. These suggested a causative relationship between uric acid and BP potentially through mediation of plasma renin activity, increased aldosterone secretion, and higher salt sensitivity.

### 3.6 Restore circadian rhythm

Several studies have adopted 24-h ambulatory BP monitoring in conjunction with office BP to appreciate the hemodynamic effects of SGLT2 inhibitors. Interestingly, SGLT2 inhibition restored the circadian rhythm of BP, which is an independent factor affecting cardiovascular mortality as demonstrated by the Ohasama study (49). Modulating sympathetic activity was proposed to be the pivotal mechanism that adjusted non-dipper to dipper type in patients with diabetes (50). Other pleiotropic effects of SGLT2 antagonization also contribute to physiological BP fluctuations (51), yet the exact mechanistic pathway requires further studies for better insight.

## 4 Future perspective

A variety of mechanisms have been hypothesized to explain the pleiotropic effect of SGLT2 inhibitors, but to which extent these pathways contributed to BP reduction is challenging to determine. Nevertheless, detailed elucidations upon the pleiotropy are still critical. The BP reduction after administration of SGLT2 inhibitors is no longer considered incidental but rather secondary to pleiotropic class effect. Future studies are required to correlate the pleiotropic pathways with observed differences in BP alterations among patients with relevant clinical backgrounds. How concomitant cardiovascular risk factors, hypertension, renal status, and left ventricular contractility interferes with the mechanisms of SGLT2 inhibitors has yet to be determined. The synergistic effect of SGLT2 inhibitors with traditional antihypertensive agents has not yet been validated clinically. Better insight into the BP alterations secondary to SGLT2 inhibition will facilitate its use in patients with cardiovascular risk factors.

## 5 Conclusion

The use of SGLT2 inhibitors is increasingly common. The impact on hemodynamics decreases BP, but the exact effect is determined by patient demographics and clinical factors. Moreover, the literature has recognized several pleiotropic mechanisms governing BP alterations via SGLT2 inhibitors, thus indicating that complex pathways are involved. These are also further inquiries into the benefit of SGLT2 inhibitors in heart failure and extended disease entities. Better understanding of the pleiotropic effects will facilitate clinical applications of SGLT2 inhibitors.

## Author contributions

T-WK and C-CH: conceptualization, methodology, formal analysis, investigation, and visualization. T-WK: writing—original draft preparation and writing—review and editing. C-CH: supervision, project administration, and funding acquisition. Both authors have read and agreed with the latest version of the manuscript.
